# The Association Between Gestational Diabetes and Microbiota in Placenta and Cord Blood

**DOI:** 10.3389/fendo.2020.550319

**Published:** 2020-10-21

**Authors:** Ning Tang, Zhong-Cheng Luo, Lin Zhang, Tao Zheng, Pianpian Fan, Yexuan Tao, Fengxiu Ouyang

**Affiliations:** ^1^Ministry of Education and Shanghai Key Laboratory of Children's Environmental Health, Xinhua Hospital, Shanghai Jiao Tong University School of Medicine, Shanghai, China; ^2^Department of Obstetrics and Gynecology, Lunenfeld-Tanenbaum Research Institute, Prosserman Center for Population Health Research, Mount Sinai Hospital, Institute of Health Policy, Management and Evaluation, Faculty of Medicine, University of Toronto, Toronto, ON, Canada; ^3^Department of Obstetrics and Gynecology, Xinhua Hospital, Shanghai Jiao Tong University School of Medicine, Shanghai, China; ^4^Department of Clinical Nutrition, Xinhua Hospital, Shanghai Jiao Tong University School of Medicine, Shanghai, China

**Keywords:** gestational diabetes mellitus (GDM), microbiota, placenta, cord blood, China

## Abstract

**Objective:** Early life is a critical period for gut microbial development. It is still controversial whether there is placental microbiota during a healthy pregnancy. Gestational diabetes mellitus (GDM) is associated with increased risk of metabolic syndrome in the offspring, and the mechanisms are unclear. We sought to explore whether microbiota in placenta and cord blood may be altered in GDM.

**Methods:** Placenta and cord blood samples were collected from eight GDM and seven euglycemic (control) term pregnancies in cesarean deliveries without evidence of clinical infections. The Illumina MiSeq Sequencing System was used to detect the microbiota based on the V3–V4 hypervariable regions of the 16S ribosomal RNA gene.

**Results:** The microbiota were detectable in all placental samples. Comparing GDM vs. controls, there were more operational taxonomic units (OTUs) (mean ± SE = 373.63 ± 14.61 vs. 332.43 ± 9.92, *P* = 0.024) and higher ACE index (395.15 ± 10.56 vs. 356.27 ± 8.47, *P* = 0.029) and Chao index (397.67 ± 10.24 vs. 361.32 ± 8.87, *P* = 0.04). The placental microbiota was mainly composed of four phyla: *Bacteroidetes, Firmicutes, Actinobacteria*, and *Proteobacteria* at the phylum level and 10 dominant genera at the genus level in both GDM and controls. Despite the dominant similarity in microbiota composition, at the OTU level, the abundance of *Ruminococcus, Coprococcus, Paraprevotella*, and *Lactobacillus* were higher, whereas *Veillonella* was lower in the placentas of GDM vs. controls. The microbiota was detected in one of the 15 cord blood samples, and its components were similar as to the corresponding placental microbiota at both phylum and genus levels suggesting placental microbiota as the potential source.

**Conclusions:** The most abundant phyla and genus of placental microbiota were similar in GDM and euglycemic pregnancies, but GDM was associated with higher diversity of placental microbiota. Further study is needed to confirm the existence of microbiota in cord blood in pregnancies without clinical infection.

## Introduction

Early life is a critical period for gut microbial development (1). Maternal environment may be the earliest microbial exposure for the fetus. Previously, the fetus' environment was considered sterile, but recent evidence indicated that bacteria may be transmitted to the fetus through maternal–fetal interface during pregnancy (2). Bacteria can be found in amniotic fluid (3, 4) and placenta (5). In addition, shared features were found among the microbiota detected in placenta, amniotic fluid, and newborn meconium in a recent study (5), suggesting that human gut microbial colonization may be initiated *in utero*. It was assumed historically that the placenta is sterile before birth, but in recent years, there is a controversy concerning whether bacteria exist in the placenta (3, 6–8), and this topic has not been well-explored. A few studies found low-density and low-diversity non-pathogenic bacteria in placenta and cord blood in healthy pregnancy (9–11), whereas another study did not find microbiota in placenta (12). The placenta and the umbilical cord serve as the bridge to connect the mother and the fetus, playing a critical role in fetal growth and development (3).

Few studies have examined microbiota in cord blood. One study did report that bacteria were detectable in 45% of cord blood samples from 20 healthy newborn delivered by cesarean section (13). Although it was commonly assumed that there were no bacteria in blood of healthy people except for bacteremia patients, blood microbiota (mostly composed of *Proteobacteria* phylum) was detected in people who had no biological signs of infection (14). In addition, the blood concentration of bacterial DNA was associated with higher risk of diabetes (14).

Gestational diabetes mellitus (GDM) is a common pregnancy complication with a prevalence ranging from 12.8 to 16.7% in China (15). GDM is characterized by elevated blood glucose levels (16) and is associated with increased risk of metabolic syndrome in the offspring (17). It remains poorly understood how the offspring of GDM harbored an increased susceptibility to metabolic syndrome–related disorders. Recent studies reported that diabetes during pregnancy might induce microbiota dysbiosis in the meconium of newborns (18–20). Considering the importance of the microbiota for metabolic health (21–23), we may speculate that alterations in the microbiota in early life may have profound impact on metabolic health in later life. It is unknown whether GDM is associated with alterations of microbiota in placenta and cord blood. We are aware of only two studies on placental microbiota in GDM (24, 25). One study reported that the proportions of *Pseudomonadales* at the order level and *Acinetobacter* at the genus level were relatively lower abundance in placenta of women with GDM (24). At the phylum level, the relative abundance of *Proteobacteria* was reported to be higher, whereas the abundance of *Bacteroidetes* and *Firmicutes* were lower in placenta of GDM pregnancy than those without GDM (25). However, further confirmation studies are needed.

In this study, we aimed to assess the existence, diversity, and composition of microbiota in placenta and cord blood in GDM and euglycemic (control) pregnancies and whether microbiota in placenta and cord blood may be altered in GDM.

## Methods

### Study Design, Participants, and Sample Collection

This was a cross-sectional study. We recruited eight GDM and seven euglycemic (control) singleton pregnant women who had prenatal care at Xinhua Hospital, a tertiary hospital in Shanghai, and were admitted to the hospital for cesarean deliveries.

Exclusion criteria were any of the followings: (1) vaginal delivery; (2) preterm birth; (3) women who had preexisting diabetes before pregnancy, or hypertension/preeclampsia, clinical chorioamnionitis, or other severe pregnancy complication; (4) women having any clinical infection or who were treated with any antibiotics during pregnancy; (5) birth defects; and (6) any antepartum infection during pregnancy. Characteristics and clinical information of study participants were obtained from medical records. The study was approved by the Research Ethics Committee of Xinhua Hospital Affiliated with Shanghai Jiao Tong University School of Medicine. Written informed consent was obtained from all participants.

Cord blood and placenta specimens were collected following a strict aseptic protocol to control potential contamination (26–28). Specifically, cord blood sample from umbilical vein was collected with a sterile injector and put into a 20-mL sterilized tube by a study obstetrician immediately after the baby birth at the delivery room. Following cesarean delivery, placenta was placed in a sterile container and then transported to a sterilized cutting-off bench in the room next to delivery room. The placenta was sampled by two study clinicians. First, the amniotic membrane was removed from surface of the fatal side of placenta with sterile scalpel and forceps. Then, four 1 × 1 × 1-cm placental biopsies were sampled from the fetal side of the placenta at each of its four quadrants with sterilized scalpel and forceps and placed in a sterile cryovial. Both cord blood and placental samples were stored at −80°C until assays.

### DNA Extraction and V3–V4 Region of 16S rRNA Gene Sequencing

Microbial DNA was extracted from placenta and cord blood samples using beads-beating method (29). Alongside the placental and cord blood samples, the background negative control was set and subjected to the same DNA extraction procedure and analysis protocol for each step of the entire experimental procedure using laboratory reagents of the same LOT number in a class II biological safety cabinet (to avoid environmental contamination). The detailed description of the methods is available in Appendix 1. Specifically, the V3–V4 region of the bacterial 16S ribosomal RNA (16S rRNA) gene in all samples (including placenta and cord blood sample and negative control sample) was amplified by polymerase chain reaction (PCR) using bar-coded universal primers F1 and R2 (5′- CCTACGGGNGGCWGCAG-3′ and 5′-GACTACHVGGGTATCTAATCC-3′) corresponding to positions 341 to 805. In the above primers, “W” represents A or T, “H” represents A, T, or C; and the “V” represents G, A, or C. During PCR reactions, blank PCR reagents were also set to avoid regents and environmental contamination. PCR reactions were carried out with the following recipe: 2 μL dNTPs (2.5 mM), 5 μL 5 × FastPfu Buffer, 0.5 μL FastPfu Polymerase, 1 μL each forward and reverse primer (5 μM), 10 ng template DNA, and PCR-grade water in a final volume of 20 μL on a GeneAmp® PCR System 9700 (Thermo Fisher Scientific Inc., Waltham, MA, USA) with the conditions: 94°C for 3 min followed 94°C for 30 s, 55°C for 30 s, 72°C for 30 s for 21 cycles, and a final extension of 8 min at 72°C (30). Amplicons were sequenced using the Illumina MiSeq platform (Illumina, San Diego, CA, USA) with paired-end 300 cycle MiSeq Reagent Kit V2 (Illumina) (31). Nuclease-free materials were used throughout the process of DNA extraction, 16S rRNA gene amplification, and library preparation, and gloves were changed between handling each sample to prevent accidental cross-contamination between samples. The laboratory technicians were blinded to the clinical status (GDM or control) of study participants.

### Analysis of 16S rRNA V3–V4 Sequence Data

High-quality sequence alignments, sequence clustering, and operational taxonomic unit (OTU) delineation were conducted based on previously established method (32). On the Quantitative Insights Into Microbial Ecology (QIIME) platform, all subsequent analyses were conducted. The analysis data were extracted from the raw data using USEARCH 8.0 (33), of which the detailed process of data clean and filtering criteria is shown in Appendix 2. OTUs were classified based on 97% similarity after the chimeric sequences were removed using UPARSE (version 7.1 http://drive5.com/uparse/). The phylogenetic affiliation of each 16S rRNA gene sequence was analyzed by RDP Classifier (http://rdp.cme.msu.edu/) against the Silva (SSU123) 16S rRNA database using the confidence threshold of 70% (34). The alpha diversity was calculated with observed OTUs, ACE index, Chao index, and Shannon index for each sample and compared between GDM and control groups using Wilcoxon signed rank test. Beta diversity was evaluated by principal coordinate analysis (PCoA) based on Bray–Curtis distance (35, 36). Sample clustering in beta diversity was tested by Adonis using R package vegan (http://www.R-project.org/). For those placental microbiota OTUs that differed between GDM and non-GDM pregnancies, the Spearman correlation coefficients were calculated with maternal clinical parameters and birth outcomes.

The relative abundances of microbiota were calculated at both phylum and genus levels in both GDM and non-GDM pregnancies. The statistical analyses on placental microbiota difference between GDM and non-GDM pregnancies used permutational multivariate analysis of variance (9,999 permutations, *P* < 0.001) with R (version 3.4.0) (32).

### Data Availability

The raw sequence data of the 16S rRNA gene supporting the results of this article are available in the NCBI Sequence Read Archive (SRA) under access number SRA958249.

### Statistical Analysis

To compare the differences in characteristics of study participants between GDM and control groups, χ^2^ or Fisher's exact test was used for categorical variables, and *t*-test or non-parametric Wilcoxon test was used for continuous variables where appropriate. All statistical analysis was carried out in SAS 9.3 software (SAS Institute, Inc., Cary, NC, USA). Two-sided *P* < 0.05 was considered statistically significant.

## Results

### Characteristics of Study Subjects

This study included eight GDM and seven euglycemic (control) pregnant women and their newborns. All study participants were urban Shanghai residents and of Han ethnicity, and 46.67% had college education or more. The clinical characteristics of mothers and newborns are shown in Table 1. There were no statistically significant differences in maternal education, parity, and prepregnancy body mass index (BMI) between women with and without GDM. Women with GDM were older than women with a euglycemic pregnancy. As expected, plasma glucose levels at fasting, 1 h, and 2 h in the 75 g oral glucose tolerance test (OGTT) and whole blood HbA_1c_ levels were all significantly higher in GDM vs. euglycemic pregnancies (Table 1) at mean gestational age of 22 to 24 weeks. All eight women with GDM received nutrition therapy and moderate exercise immediately after the diagnosis of GDM. None of them were treated with insulin. All newborns were term born singletons without fetal growth restriction. There were no differences in gestational age at delivery or birth weight comparing GDM vs. non-GDM pregnancies.

**Table 1:** Characteristics of study mother-newborn pairs.

	**Non-GDM (*n* = 7)**	**GDM (*n* = 8)**	P^*^
**Mothers**			
Maternal age (years)	31.2 ± 3.8	35.4 ± 2.7	0.03
Education, collage or more	4 (57.14%)	4 (50.00%)	1.00
Education, under collage	3 (42.86%)	4 (50.00%)	
Primiparous	2 (28.57%)	4 (50.00%)	0.61
Prepregnancy BMI (kg/m^2^)	22.5 ± 2.1	25.0 ± 2.3	0.05
GA at 75 g OGTT (weeks)	24.4 ± 1.3	22.0 ± 3.6	0.09
**Plasma glucose in OGTT (mmol/L)**			
Fasting	4.4 ± 0.2	5.1 ± 0.5	0.01
1 h	7.3 ± 0.5	9.3 ± 0.6	<0.0001
2 h	5.4 ± 1.0	7.4 ± 1.1	0.0003
HbA_1c_ (%)	4.9 ± 0.2	5.5 ± 0.2	0.0003
**Late pregnancy (35–38 weeks)**			
Fasting plasma glucose (mmol/L)	4.2 ± 0.3	4.8 ± 0.2	0.0009
Serum triglycerides (mmol/L)	2.2 ± 0.7	4.6 ± 3.0	0.06
Serum total cholesterol (mmol/L)	6.2 ± 0.6	6.5 ± 0.8	0.42
**Newborns**			
Gestational age (weeks)	39.0 ± 0.6	39.3 ± 0.3	0.21
Birth weight (g)	3734.3 ± 339.9	3517.5 ± 361.5	0.23
Birth length (cm)	50.29 ± 0.95	49.75 ± 1.28	0.38
Ponderal index	2.93 ± 0.19	2.85 ± 0.24	0.49
Sex, male	6 (85.71%)	2 (25.00%)	0.04

*Data presented are mean ± SD or n (%)*.

* *P-values for comparisons between the 2 groups in t-tests for continuous variables, and χ^2^ and Fisher's exact tests for categorical variables*.

### Characteristics of 16S rRNA Gene Sequencing in Placenta and Cord Blood Samples

Bacteria 16S rRNA can be detected in all of the 15 placental specimens, but in only one of the 15 cord blood specimens. Negative control sample from DNA isolation and blank PCR reagent were both negative after PCR step, which was considered as having no contamination from reagents and environment.

Overall, 622,263 high-quality sequences were obtained in the 15 placental samples. The average number was 41,484.2 per placental sample. The Good's coverage of each sample was more than 97%, indicating that the sequences identified can represent the majority of bacteria in each of the placental samples. In one cord blood sample, 49,569 high-quality sequences were obtained.

### The Diversity of Placenta Microbiota in GDM and Non-GDM Pregnancies

**Figure 1:**
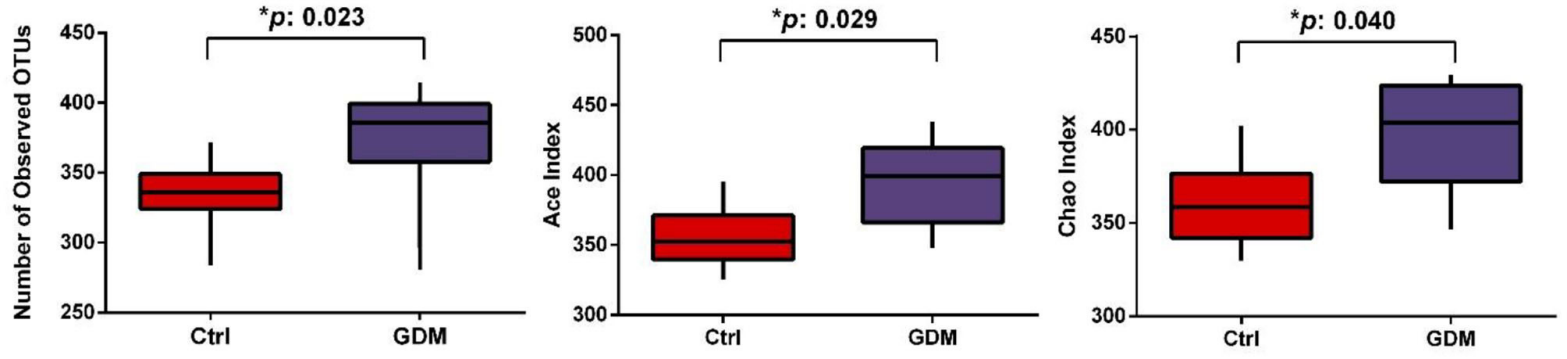
The diversity of placental microbiota in women with GDM (*n* = 8) compared to women without GDM (control, *n* = 7) at the operational taxonomic unit (OTU) level.

The mean number of OTUs was 373.63 (SE, 14.61) in the placentas of women with GDM, higher than that of control women (332.43 ± 9.92, *P* = 0.024). Comparing GDM vs. control groups, the ACE indices were higher (395.15 ± 10.56 vs. 356.27 ± 8.47, *P* = 0.029), and Chao indices were also higher (397.67 ± 10.24 vs. 361.32 ± 8.87, *P* = 0.040) in GDM (Figure 1), indicating that placental microbiota in GDM pregnancies had greater diversity and community richness at OTU level. The Shannon index (a bacterial community diversity indicator) was slightly higher in GDM vs. control groups (0.066 vs. 0.058, *P* = 0.054).

**Figure 2:**
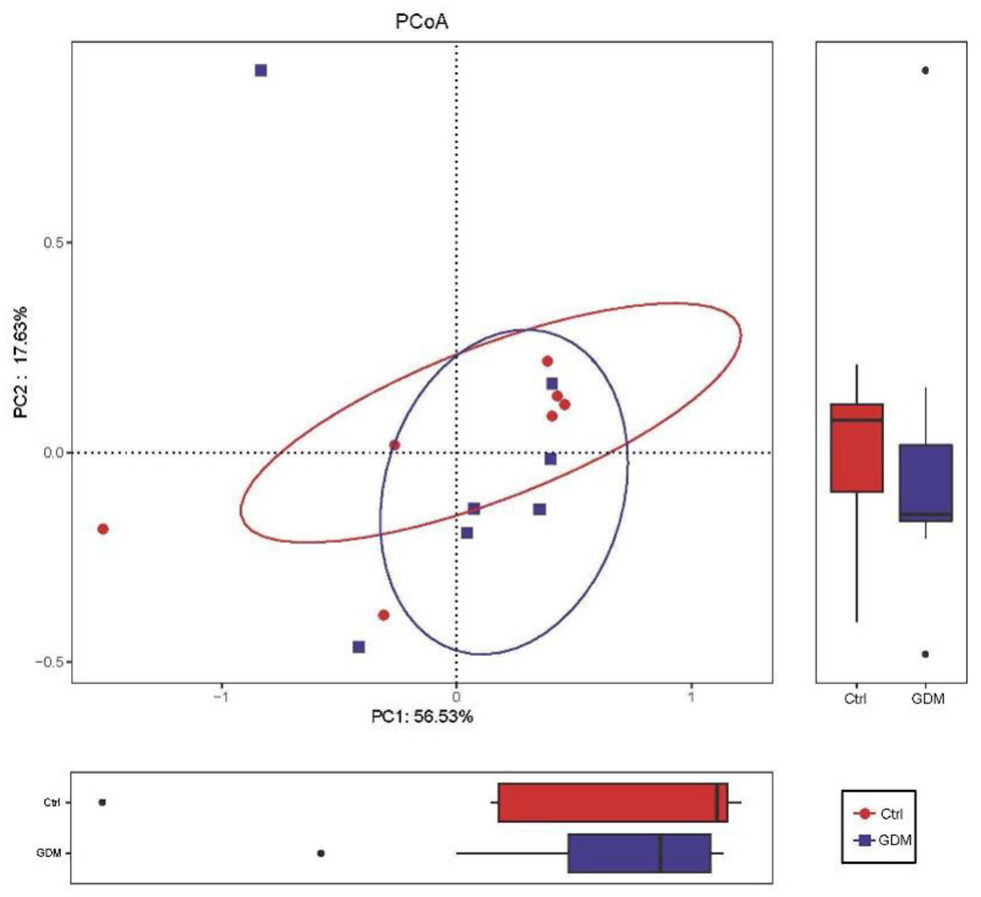
Placental microbiota structure in GDM and non-GDM women by using principal coordinate analysis (PCoA) based on Bray–Curtis distance.

To compare overall placenta microbiota composition between GDM and euglycemic pregnancies, PCoA analysis based on Bray–Curtis distance according to OTUs of each sample was implemented. Overall, in the total variation of all placental samples, the first principal component (PC1) could account for 56.53%, and PC2 could account for 17.63%. As shown in Figure 2, the PCoA revealed no difference between GDM and control pregnancies in placental bacterial composition (*P*-value for the PCoA analysis is 0.383).

### The Composition of Placenta Microbiota in GDM and Non-GDM Pregnancies

**Figure 3:**
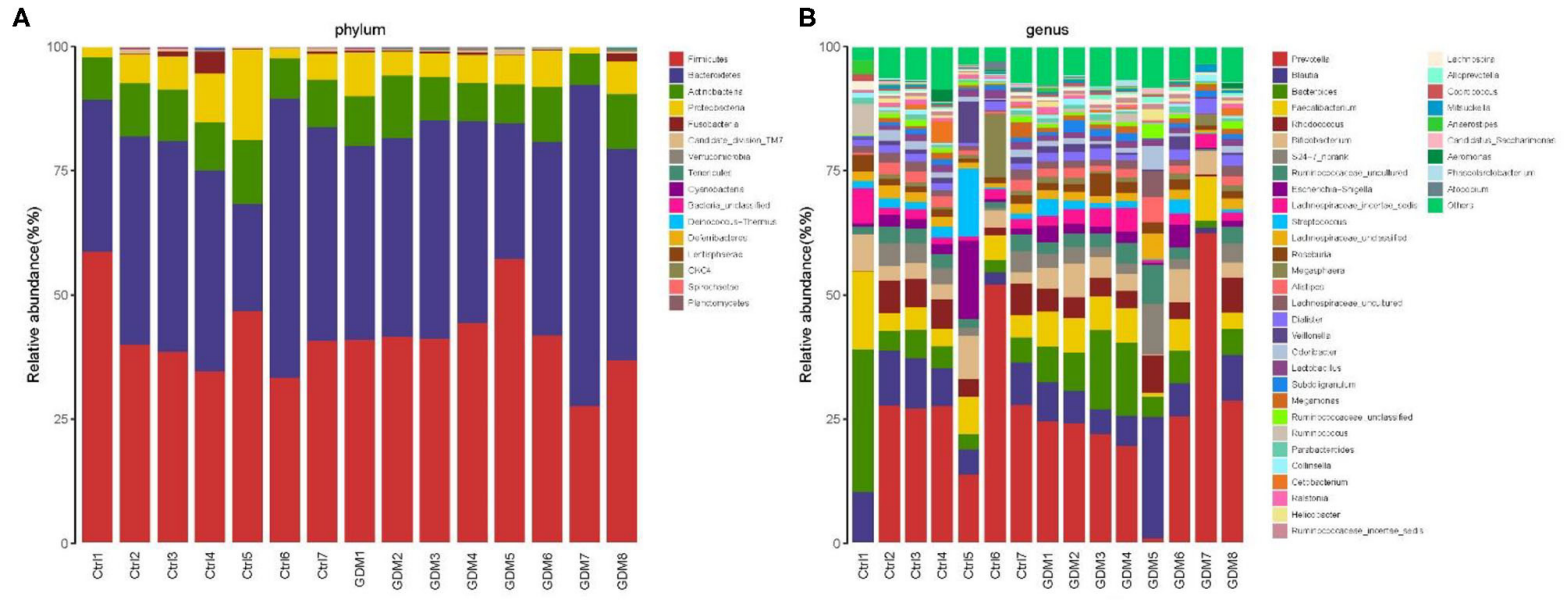
Composition of placental microbiota in eight women with GDM and seven women without GDM (control) at phylum **(A)** and genus **(B)** levels.

At the phylum level, the placenta microbiota was mainly composed of four phyla, and the relative abundances for each of 4 phyla were comparable in GDM and control groups (mean ± SE): *Bacteroidetes* (42.1 ± 3.7% in GDM vs. 39.4 ± 4.1% in control; *P* = 1.00), *Firmicutes* (41.5 ± 2.9% vs. 41.9 ± 3.3%; *P* = 0.61), *Actinobacteria* (9.4 ± 0.7% vs. 10.0 ± 0.6% in control; *P* = 0.69), and *Proteobacteria* (5.6 ± 0.8% vs. 7.1 ± 2.1%; *P* = 0.78) (Figure 3A and Table 2).

**Table 2:** Relative abundances (%) of placental microbiota at phylum and genus levels in eight women with GDM and seven women without GDM (non-GDM).

	**Non-GDM (%)**	**GDM (%)**	***P-*value**
	**Mean ± SE**	**Mean ± SE**	
**Phylum level**			
Firmicutes	41.9 ± 3.3	41.5 ± 2.9	0.61
Bacteroidetes	39.4 ± 4.1	42.1 ± 3.7	1.00
Actinobacteria	10.0 ± 0.6	9.4 ± 0.7	0.69
Proteobacteria	7.1 ± 2.1	5.6 ± 0.8	0.78
**Genus level**			
Prevotella	25.23 ± 5.99	26.07 ± 6.00	0.69
Blautia	7.90 ± 1.19	8.33 ± 2.44	0.46
Bacteroides	7.62 ± 3.52	7.85 ± 1.78	0.28
Faecalibacterium	6.33 ± 1.64	5.89 ± 0.90	0.78
Rhodococcus	4.26 ± 0.97	4.30 ± 0.79	1.00
Bifidobacterium	4.39 ± 0.95	4.16 ± 0.73	0.78
S24-7_norank	2.59 ± 0.70	3.30 ± 1.06	1.00
Ruminococcaceae_uncultured	2.44 ± 0.36	3.19 ± 0.77	0.69
Escherichia-Shigella	3.51 ± 2.05	1.94 ± 0.52	1.00
Lachnospiraceae_incertae_sedis	2.34 ± 0.80	2.57 ± 0.46	0.34

At the genus level, the placental microbiota was mainly composed of 10 dominant genus in both women with and without GDM. The relative abundances of each of the 10 genera were comparable between GDM and control pregnancies: genera (mean ± SE) *Prevotella* (26.07% ± 6.00% in GDM vs. 25.23% ± 5.99% in control), *Blautia* (8.33% ± 2.44% vs. 7.90% ± 1.19%), *Bacteroides* (7.85% ± 1.78% vs. 7.62% ± 3.52%), *Faecalibacterium* (5.89% ± 0.90% vs. 6.33% ± 1.64%), *Rhodococcus* (4.30% ± 0.79% vs. 4.26% ± 0.97%), *Bifidobacterium* (4.16% ± 0.73% vs. 4.39% ± 0.95%), *S24.7_norank* (3.30% ± 1.06% vs. 2.59% ± 0.70%), *Ruminococcaceae_uncultured* (3.19% ± 0.77% vs. 2.44% ± 0.36%), *Escherichia.Shigella* (1.94% ± 0.52% vs. 3.51% ± 2.05%), and *Lachnospiraceae_incertae_sedis* (2.57% ± 0.46% vs. 2.34% ± 0.80%) (Figure 3B and Table 2).

**Figure 4:**
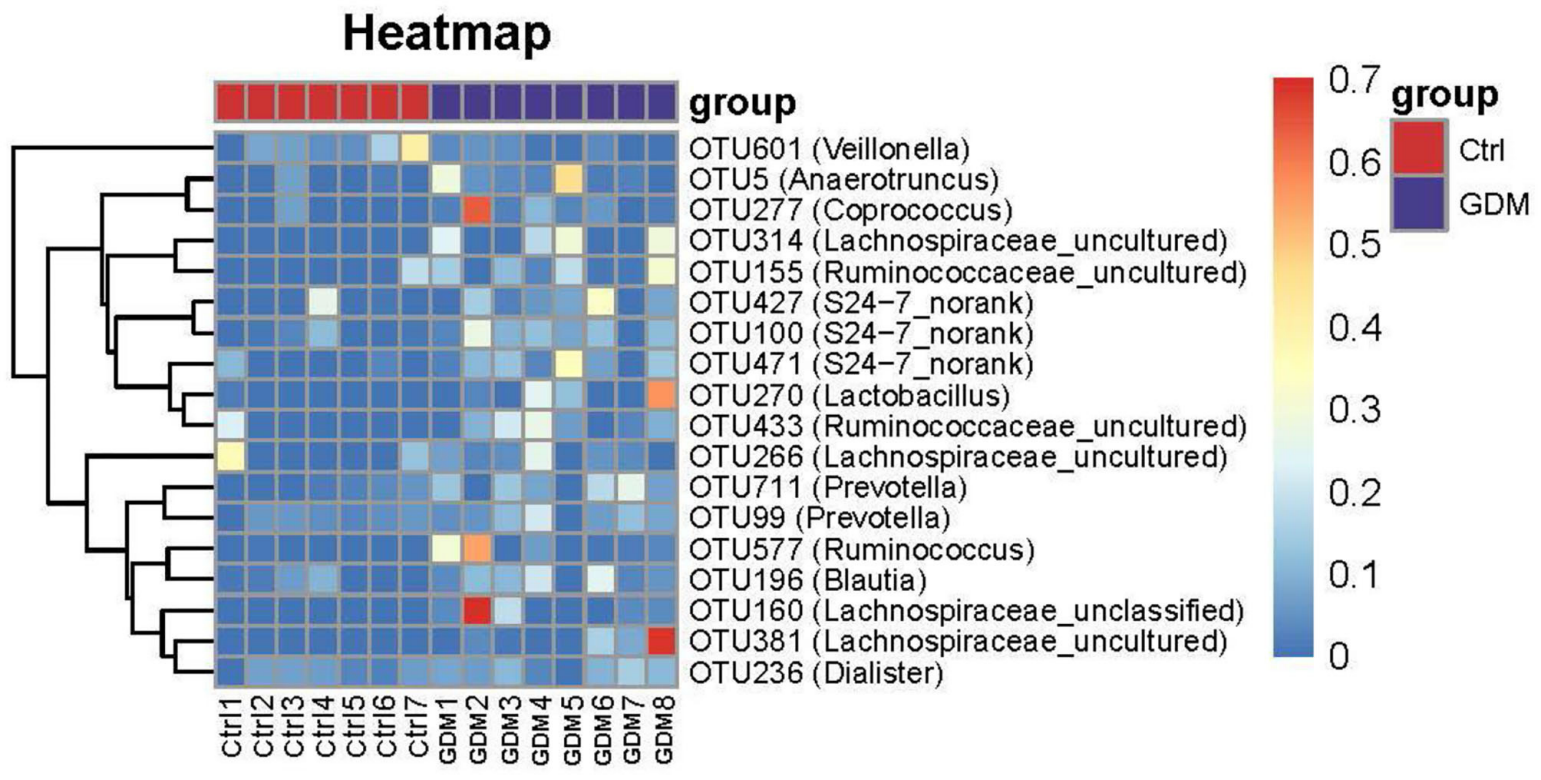
Key OTU heat map analyses of placental bacteria in women with GDM (*n* = 8) and those without GDM (*n* = 7).

Despite above similarity, this study also identified 18 OTUs of placental microbiota that differed between GDM and non-GDM pregnancies (Figure 4 and Table 3). The relative abundance of *Coprococcus* (OTU 277), *Prevotella* (OTU 711), *Anaerotruncus* (OTU 5), *S24–7_norank* (OTU 100, 471), *Ruminococcus* (OTU 577, 495), *Ruminococcaceae_uncultured* (OTU 155, 509, 495), *Lachnospiraceae_uncultured* (OTU 381, 314, 665), *Lachnospiraceae_unclassified* (OTU 160, 41), *Paraprevotella* (OTU 415), *Dorea* (OTU 588), and *Defluviitaleaceae_Incertae_Sedis* (OTU 494) were all higher, whereas *Veillonella* (OTU 601) was lower in the placentas of women with GDM than those of controls (Figure 4 and Table 3). Table 3 shows a list of the predominant microbiota (*n* = 18) at the OTU level that differed between the GDM and control groups (*P* < 0.05).

**Table 3:** Relative abundances (%) of placental microbiota OTUs that differed between eight women with GDM and seven women without GDM (non-GDM).

**OTUs**	**Non-GDM**		**GDM**		***P-*value**
	**Mean**	***SD***	**Mean**	***SD***	
OTU277	10.0 × 10^−3^	2.2 × 10^−3^	9.0 × 10^−3^	1.7 × 10^−1^	0.013
*(Coprococcus)*					
OTU160	0	0	9.6 × 10^−3^	1.8 × 10^−3^	0.019
*(Lachnospiraceae_unclassified)*					
OTU381	0	0	1.7 × 10^−3^	3.2 × 10^−3^	0.019
*(Lachnospiraceae_uncultured)*					
OTU601	1.3 × 10^−1^	1.6 × 10^−1^	2.7 × 10^−2^	2.7 × 10^−2^	0.027
*(Veillonella)*					
OTU494	1.4 × 10^−2^	2.3 × 10^−2^	5.8 × 10^−2^	3.6 × 10^−2^	0.029
*(Defluviitaleaceae_Incertae_Sedis)*					
OTU5	3.3 × 10^−3^	7.3 × 10^−3^	3.0 × 10^−2^	4.4 × 10^−2^	0.031
*(Anaerotruncus)*					
OTU588	2.7 × 10^−2^	3.5 × 10^−2^	9.5 × 10^−2^	6.3 × 10^−2^	0.032
*(Dorea)*					
OTU155	6.7 × 10^−3^	1.8 × 10^−2^	2.5 × 10^−2^	2.8 × 10^−2^	0.033
*(Ruminococcaceae_uncultured)*					
OTU711	7.8 × 10^−3^	8.6 × 10^−3^	4.7 × 10^−2^	3.8 × 10^−2^	0.036
*(Prevotella)*					
OTU577	7.9 × 10^−4^	9.8 × 10^−4^	3.2 × 10^−2^	5.1 × 10^−2^	0.036
*(Ruminococcus)*					
OTU509	3.0 × 10^−3^	3.3 × 10^−3^	4.1 × 10^−2^	4.8 × 10^−2^	0.042
*(Ruminococcaceae_uncultured)*					
OTU471	1.9 × 10^−3^	3.8 × 10^−3^	9.6 × 10^−3^	9.9 × 10^−2^	0.042
*(S24-7_norank)*					
OTU415	3.7 × 10^−4^	9.8 × 10^−4^	1.4 × 10^−2^	2.8 × 10^−2^	0.043
*(Paraprevotella)*					
OTU495	4.9 × 10^−4^	1.3 × 10^−3^	1.4 × 10^−2^	1.8 × 10^−2^	0.043
*(Ruminococcaceae_uncultured)*					
OTU41	0	0	1.0 × 10^−2^	1.5 × 10^−2^	0.045
*(Lachnospiraceae_unclassified)*					
OTU314	0	0	1.9 × 10^−2^	2.1 × 10^−2^	0.045
*(Lachnospiraceae_uncultured)*					
OTU665	0	0	1.4 × 10^−2^	1.6 × 10^−2^	0.045
*(Lachnospiraceae_uncultured)*					
OTU100	7.6 × 10^−3^	1.4 × 10^−2^	3.4 × 10^−2^	2.7 × 10^−2^	0.047
*(S24-7_norank)*					

### Correlation Between Relative Abundances of Above 18 Altered Placenta Microbiota OTUs and Clinical Characteristics of Mothers and Neonates

**Figure 5:**
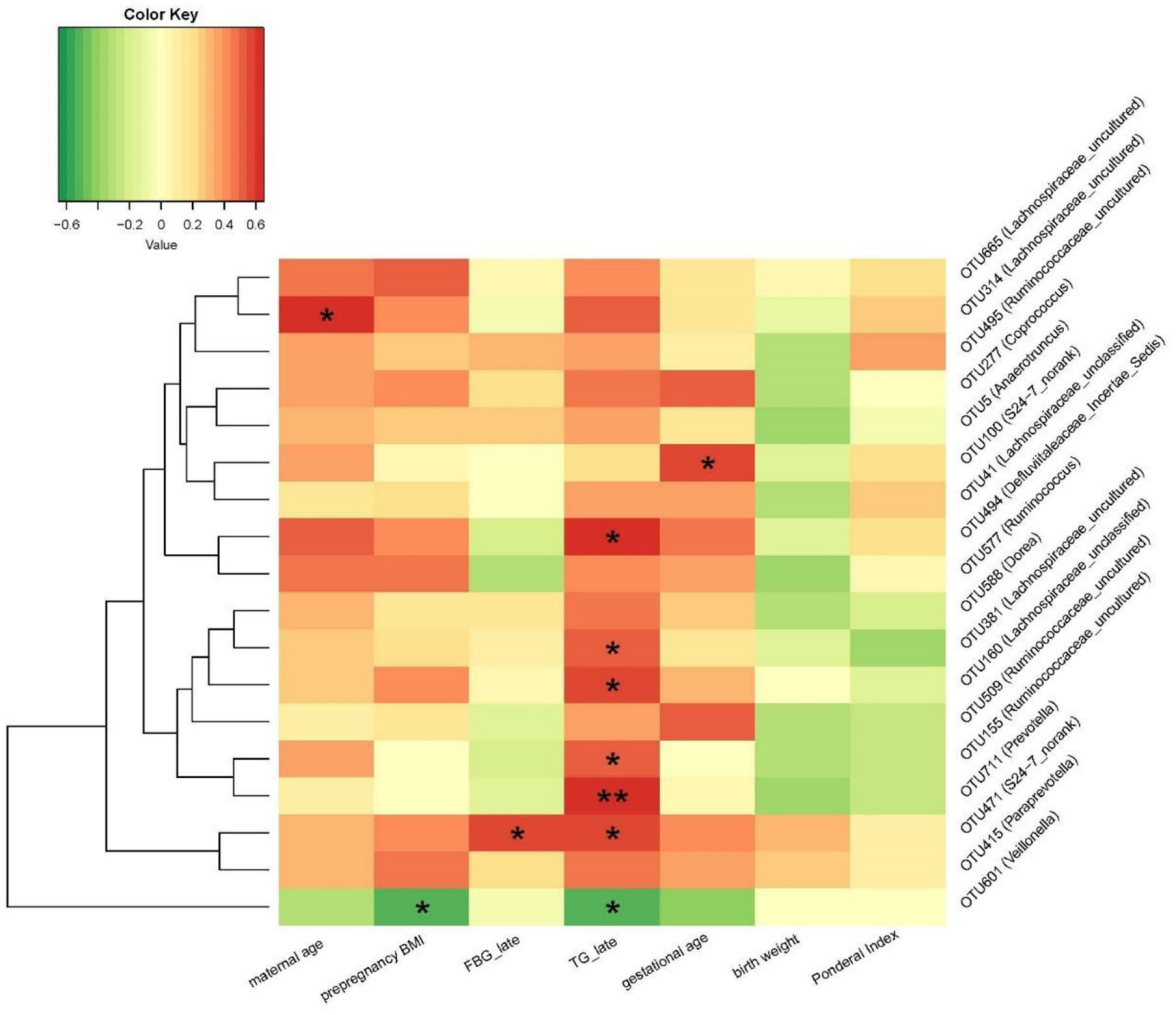
Correlation between the relative abundance of altered placenta microbiota at OTU level and clinical characteristics in mothers and neonates. *P < 0.05, **P < 0.01. OTU, operational taxonomic unit; FBG, fasting blood glucose; TG, triglycerides.

Figure 5 shows the Spearman correlation between the relative abundances of 18 altered placenta microbiota OTUs (between GDM and non-GDM pregnancies) and specific clinical characteristics of mothers and infants. The relative abundance of OTU 314 (*Lachnospiraceae_uncultured*) was positively correlated with maternal age. OTU 100 (*S24-7_norank*) was positively correlated with gestational age. OUT 471 (*S24-7_norank*) was positively correlated with fasting blood glucose in late pregnancy; OTU 471 (*S24-7_norank)*, OTU 711 (*Prevotella)*, OTU 155 (*Ruminococcaceae_uncultured)*, OTU 160 (*Lachnospiraceae_unclassified)*, OTU 381 (*Lachnospiraceae_uncultured*), and OTU 494 (*Defluviitaleaceae_Incertae_Sedis*) were positively correlated with maternal serum triglycerides concentration at late pregnancy. OTU 601 (*Veillonella*) was negatively correlated with maternal serum triglycerides concentration at late pregnancy and prepregnancy BMI. None of the OTUs was correlated with infant birth weight and Ponderal index (Figure 5).

### The Microbiota in Cord Blood

**Figure 6:**
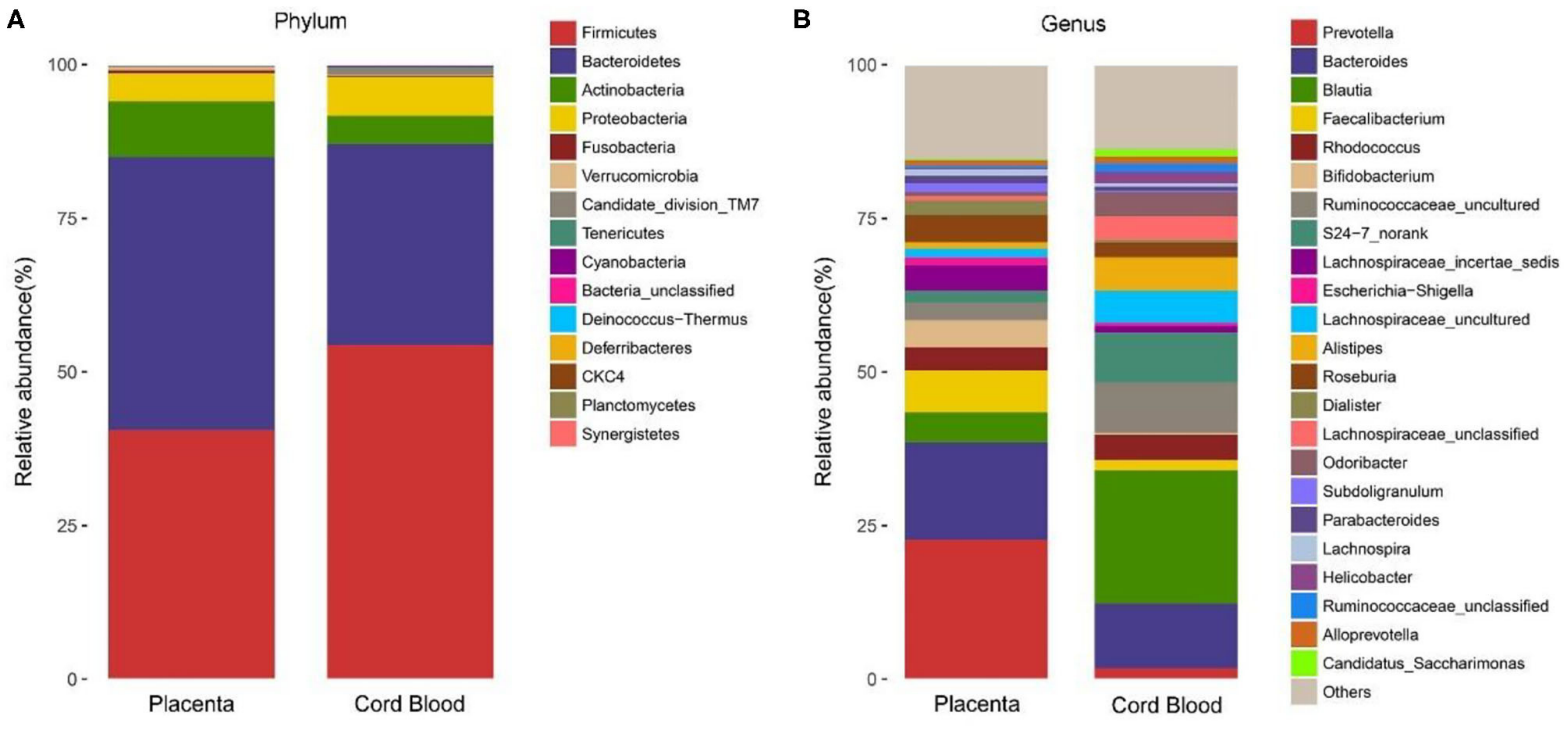
The microbiota composition detected in one cord blood sample and in its corresponding placenta from the same GDM woman at phylum **(A)** and genus **(B)** levels.

The microbiota of cord blood was detectable in only one of the 15 samples, from a woman with GDM, and the number of OTUs was 415. The microbiota at the phylum level were mainly composed of *Firmicutes* (54.4%), *Bacteroidetes* (32.7%), *Proteobacteria* (6.3%), and *Actinobacteria* (4.6%). The microbiota at the genus level were mainly composed of *Blautia* (21.6%), *Bacteroides* (10.5%), *Ruminococcaceae_uncultured* (8.2%), *S24-7_norank* (8.1%), *Alistipes* (5.4%), *Lachnospiraceae_uncultured* (5.2%), and *Rhodococcus* (4.1%). At both phylum and genus levels, the detectable bacteria in the cord blood sample were similar as those detected in the corresponding placental sample from the same woman (Figure 6).

## Discussion

In this study, microbiota was detectable in all the placental samples and in one of 15 cord blood samples. The placental microbiota of GDM pregnancies had higher diversity, as evidenced by more OTUs and higher ACE and Chao indices than those of euglycemic pregnancies. The placental microbiota was mainly composed of four most abundant phyla including *Bacteroidetes, Firmicutes, Actinobacteria*, and *Proteobacteria* in both GDM and euglycemic women. Still, 18 OTUs were found to be significantly different in relative abundance between GDM and control subjects, with a higher abundance of *Ruminococcus, Coprococcus, Paraprevotella, Lactobacillus, S24–7, Lachnospiraceae, Anaerotruncus, Defluviitaleaceae, Dorea*, and *Blautia* OTUs and a lower abundance of *Veillonella* OTU in GDM.

There is some controversy concerning whether the placenta without clinical infection contains microbiota. Most existing studies reported that placentas harbored the microbiota (9, 11, 24, 25, 37). These studies showed that the placenta microbiota at phylum level was dominantly composed of *Proteobacteria, Bacteroidetes, Firmicutes, Actinobacteria*, and *Cyanobacteria* phylum (9, 11, 24, 25, 37), which was consistent with the results in this study. Among these studies, we are aware of only two small studies on placental microbiota in GDM pregnancies (24, 37). One study (24) observed that the number of OTUs was higher for placenta microbiota in GDM (*n* = 10 GDM and 10 controls), which was consistent with our study finding. In contrast, another study reported reduced OTUs in placenta microbiota of GDM pregnancies (*n* = 11 GDM and 11 controls) (37). Some studies argued that healthy placentas had no microbiota (6, 7, 38, 39). Previous studies emphasize that negative control is important (28), and sterile technical precautions during sample collection and processing are necessary. In our study, we set background negative control, which was subjected to the same DNA extraction procedure and analysis protocol alongside the placental and cord blood samples as quality control. We detected microbiota in all placental samples but not in the negative control sample. The association between GDM and microbiota has been studied in feces and meconium microbiota in recent years (18, 19, 40, 41). A study reported that the gut microbiota of women with GDM had a higher α-diversity and higher relative abundance of *Firmicutes* and lower relative abundance of *Bacteroidetes* and *Actinobacteria* phylum (40). A recent study found that microbiota in salivary samples had lower abundance at OTU level in GDM women during late pregnancy and postpartum period, suggesting reduced diversity (42). Our finding in the placentas is in agreement with the previous finding on GDM and gut microbiota (40).

The abundance of *Bacteroides* in placental tissue has been associated with the expression of genes that were important for nutrient transport and immunity in pregnant women' placenta (43). *Bacteroides* species in gut microbiota increased especially in obesity women and may transfer to the babies during vaginal delivery (43–45). In the present study, the *Bacteroides* genus was detected in placenta of both GDM and non-GDM. A previous study showed that *Ruminococcus* participated in the phosphorylate process of cellobiose and glucose (46). Another study found that *Coprococcus* fermented polysaccharides and produced short-chain fatty acids such as butyric acid and acetic acid in human fecal samples (47). We speculated that higher glucose levels in GDM might partly account for the higher abundance of *Ruminococcus* and *Coprococcus* at OTU level in GDM.

Compared to those in healthy pregnant women, the compositions of oral, vaginal, and rectal microbiota are different in GDM patients, respectively (48). It has not been fully explored how maternal GDM influences microbiota in placenta. GDM is characterized by elevated blood glucose (16), and studies suggest that placenta exposed to hyperglycemic milieu had increased inflammation, oxidative stress, and angiogenesis of fetoplacental vessels (49). The origin of placenta microbiota has not been fully explored. Recent studies suggest that three potential routes might exist during pregnancy: the oral–placental, gastrointestinal/gut–placental, and genitourinary/vaginal–placental routes (50). The placental microbiota was mostly similar as oral microbiota and can be transmitted from oral bacteria *via* hematogenous spread (9). Second, microbes can be translocated from the maternal gut into the lymphatic system *via* dendritic cells and then delivered to the placenta *via* maternal bloodstream (51). Third, microorganisms may ascend from the vagina to the placenta due to anatomical location adjacent (10). However, it is still not entirely clear how microorganisms enter placental and fetal compartment. The maternal lipid level in blood was associated with GDM; triglyceride levels were increasing in women with GDM compared with those euglycemic pregnancies (52). In this study, we found that some OTUs in placenta microbiota were positively correlated with triglycerides in late pregnancy, and this indicates that triglycerides may participate in the metabolism of the flora in gut (53, 54). *Lachnospiraceae_uncultured* was positively correlated with maternal age. In rabbit study, *Lachnospiraceae* family in gut was also found increasing with age and became the dominant taxa at 80 days (55).

Studies on microbiota in cord blood were scarce. In 2005, Jimenez et al. isolated bacteria from cord blood in healthy newborns born by C-section, and they detected bacteria in 45% (9/20) of cord blood samples (13). In 2011, Amar et al. reported that the higher concentrations of bacterial 16S rDNA in venous blood were associated with higher risk of diabetes in 6–9 years later. Another recent study also found that the genus *Bacteroides* in blood was negatively associated with risk of type 2 diabetes mellitus (T2DM), whereas le there was a positive association between the genus *Sediminibacterium* and T2DM (56). In our study, we collected matched placenta and cord blood samples from the same women, which allowed us to identify if the placenta microbiota was the source for microbiota detected in cord blood sample. We detected microbiota in one of 15 cord blood samples, and the structure and composition were similar to the corresponding placental microbiota from the same woman, a possible source. Further study is needed to confirm the existence of bacteria in cord blood in healthy subjects without infections.

The main study limitation is the relatively small sample size. The preliminary finding requires confirmation in larger studies. Importantly, our study revealed the differences in the placental microbiota between GDM and non-GDM pregnancies at the OTU level. In this study, we did not collect the exact housing and living situation of each woman in the study. Housing characteristics (for example, number of children/people) may have an impact on household microbial diversity (57). Living environment and socioeconomic status may also affect the composition of gut microbiota (58–61), as gut microbial communities can be shaped by diet (58) and lifestyle (60). However, there was no difference in maternal education level between the GDM and normal groups. Also, this is a homogenous urban population, and all women were of Han ethnicity and thus had relatively similar lifestyle and dietary habit.

In conclusion, this study confirmed that microbiota existed in the placenta and had higher diversity in GDM compared with euglycemia pregnancy. Future studies are needed to uncover the roles of placental microbiota for long-term metabolic health in children.

## Data Availability Statement

The raw sequence data of the 16S rRNA gene supporting the results of this article are available in the NCBI Sequence Read Archive (SRA) under access number SRA958249.

## Ethics Statement

The studies involving human participants were reviewed and approved by Research Ethics Committee of Xinhua Hospital Affiliated with Shanghai Jiao Tong University School of Medicine. The patients/participants provided their written informed consent to participate in this study.

## Author Contributions

FO conceived and designed the study. FO, NT, LZ, TZ, and YT coordinated and conducted the study. FO and NT analyzed, interpreted data, and drafted the manuscript. Z-CL and PF intensively reviewed and revised the manuscript. All authors have approved the final version of the manuscript. All authors contributed to the article and approved the submitted version.

## Conflict of Interest

The authors declare that the research was conducted in the absence of any commercial or financial relationships that could be construed as a potential conflict of interest.
